# COVID-19 induces new-onset insulin resistance and lipid metabolic dysregulation via regulation of secreted metabolic factors

**DOI:** 10.1038/s41392-021-00822-x

**Published:** 2021-12-16

**Authors:** Xi He, Chenshu Liu, Jiangyun Peng, Zilun Li, Fang Li, Jian Wang, Ao Hu, Meixiu Peng, Kan Huang, Dongxiao Fan, Na Li, Fuchun Zhang, Weiping Cai, Xinghua Tan, Zhongwei Hu, Xilong Deng, Yueping Li, Xiaoneng Mo, Linghua Li, Yaling Shi, Li Yang, Yuanyuan Zhu, Yanrong Wu, Huichao Liang, Baolin Liao, Wenxin Hong, Ruiying He, Jiaojiao Li, Pengle Guo, Youguang Zhuo, Lingzhai Zhao, Fengyu Hu, Wenxue Li, Wei Zhu, Zefeng Zhang, Zeling Guo, Wei Zhang, Xiqiang Hong, Weikang Cai, Lei Gu, Ziming Du, Yang Zhang, Jin Xu, Tao Zuo, Kai Deng, Li Yan, Xinwen Chen, Sifan Chen, Chunliang Lei

**Affiliations:** 1 Guangzhou Eighth People’s Hospital, Guangzhou Medical University, Guangzhou, China; 2 Division of Vascular Surgery, The First Affiliated Hospital of Sun Yat-sen University, Guangzhou, China; 3 National-Guangdong Joint Engineering Laboratory for Diagnosis and Treatment of Vascular Diseases, The First Affiliated Hospital of Sun Yat-sen University, Guangzhou, China; 4 Guangdong Provincial Key Laboratory of Malignant Tumor Epigenetics and Gene Regulation, Guangdong-Hong Kong Joint Laboratory for RNA Medicine, Sun Yat-Sen Memorial Hospital, Sun Yat-Sen University, Guangzhou, China; 5 Medical Research Center, Sun Yat-Sen Memorial Hospital, Sun Yat-Sen University, Guangzhou, China; 6 Department of Obstetrics and Gynecology, Guangzhou Women and Children Medical Center, Guangzhou Medical University, Guangzhou, China; 7 Institute of Human Virology, Key Laboratory of Tropical Disease Control of Ministry of Education, Zhongshan School of Medicine, Sun Yat-sen University, Guangzhou, China; 8 Department of Immunology, Zhongshan School of Medicine, Sun Yat-sen University, Guangzhou, China; 9 Guangzhou Center for Disease Control and Prevention, Guangzhou, China; 10 Wuhan Metware Biotechnology Co., Ltd, Wuhan, China; 11 Department of Biomedical Sciences, New York Institute of Technology, College of Osteopathic Medicine, Old Westbury, NY USA; 12 Max Planck Institute for Heart and Lung Research and Cardiopulmonary Institute (CPI), Bad Nauheim, Germany; 13 Department of Molecular Diagnostics, Sun Yat-sen Cancer Center, Sun Yat-sen University, Guangzhou, China; 14 School of Public Health (Shenzhen), Sun Yat-sen University, Shenzhen, China; 15 State Key Laboratory of Biocontrol, School of Life Sciences, Sun Yat-Sen University, Guangzhou, China; 16 The Sixth Affiliated Hospital of Sun Yat-sen University, Guangzhou, China; 17 Department of Endocrinology, Sun Yat-Sen Memorial Hospital, Sun Yat-sen University, Guangzhou, China; 18 Guangzhou Regenerative Medicine and Health-Guangdong Laboratory (GRMH-GDL), Guangzhou Institutes of Biomedicine and Health, Chinese Academy of Sciences, Guangzhou, China; 19 Key Laboratory of Regenerative Biology of the Chinese Academy of Sciences and Guangdong Provincial Key Laboratory of Stem Cell and Regenerative Medicine, Guangzhou Institutes of Biomedicine and Health, Chinese Academy of Sciences, Guangzhou, China

**Keywords:** Endocrine system and metabolic diseases, Infectious diseases

## Abstract

Abnormal glucose and lipid metabolism in COVID-19 patients were recently reported with unclear mechanism. In this study, we retrospectively investigated a cohort of COVID-19 patients without pre-existing metabolic-related diseases, and found new-onset insulin resistance, hyperglycemia, and decreased HDL-C in these patients. Mechanistically, SARS-CoV-2 infection increased the expression of RE1-silencing transcription factor (REST), which modulated the expression of secreted metabolic factors including myeloperoxidase, apelin, and myostatin at the transcriptional level, resulting in the perturbation of glucose and lipid metabolism. Furthermore, several lipids, including (±)5-HETE, (±)12-HETE, propionic acid, and isobutyric acid were identified as the potential biomarkers of COVID-19-induced metabolic dysregulation, especially in insulin resistance. Taken together, our study revealed insulin resistance as the direct cause of hyperglycemia upon COVID-19, and further illustrated the underlying mechanisms, providing potential therapeutic targets for COVID-19-induced metabolic complications.

## Introduction

Coronavirus disease 2019 (COVID-19) has spread worldwide and resulted in 251,266,207 confirmed cases, with the death toll rising to 5,070,244 as to November 10, 2021 (https://covid19.who.int/). This pandemic has overwhelmingly impeded social-economic conditions and healthcare services around the world.

A number of key comorbidities, including diabetes, obesity, hypertension, and cardiovascular diseases, are associated with worse prognosis in patients with COVID-19.^1–4^ Zhou et al. reported higher mortality rates in COVID-19 patients with diabetes and hypertension.^2^ Whereas, BMI > 40 kg/m^2^ was the second strongest independent predictor of hospitalization among 4103 patients with COVID-19 admitted to an academic health system.^5^ Furthermore, Wu et al. reported that patients with cardiovascular disease had the highest case-fatality rate of 10.5% among those with pre-existing comorbid conditions,^6^ suggesting that cardiovascular metabolic comorbidities might drive the progression and deteriorate clinical outcomes of COVID-19.

On the other hand, a few case reports and observational studies reported abnormal glucose and lipid metabolism in COVID-19 patients recently. In detail, several observational studies reported hyperglycemia on admission in 11.9 to 28.4% of COVID-19 patients,^7–11^ as well as a decrease of high-density lipoprotein–cholesterol (HDL-C).^12–14^ Though hyperglycemia was observed in COVID-19 patients regardless of different ethnicities, ages, and genders, the underlying mechanism remains unclear. One hypothesis was that SARS-CoV-2 attacked the pancreas given the abundant expression of ACE2 in islet capillaries,^15^ which may result in acute pancreatitis with deficiency of insulin secretion. Indeed, some observational studies showed elevated plasma pancreatic enzyme (amylase and lipase) in up to 31% of COVID-19 patients, and autopsy from the COVID-19 patients showed necrosis and hemorrhage in the pancreas.^16^ Alongside the pancreas injury, type 1 diabetes potentially induced by COVID-19 was also reported with hyperglycemia, ketoacidosis, reduced C-peptide, and negative autoantibody.^17^ On the other hand, Montefuscono et al. reported associated hyperinsulinemia in COVID-19 patients,^18^ and higher prevalence of elevated C-peptide in acute respiratory distress syndrome (ARDS) with COVID-19 in comparison with COVID-19-negative ARDS patients was also reported.^19^ These studies suggested another potential hypothesis that COVID-19 may cause insulin resistance, resulting in hyperglycemia. Therefore, it is still highly debatable whether insulin deficiency or insulin resistance drives the progress of hyperglycemia in COVID-19, and more clinical data and mechanistic studies are required.

In order to investigate the metabolic dysregulation in COVID-19, several omics studies were conducted, showing significant alterations of lipids^20–22^ and proteins^22–26^ in the sera of COVID-19 patients, which might potentially shed light on the illustration of mechanism regarding COVID-19-associated metabolic dysregulation. However, these omics studies might be biased due to the comorbidity of diabetes, nonalcoholic fatty liver disease, or other metabolic disorders in some patients included. Moreover, the interpretation of the alteration of secreted metabolic proteins in COVID-19 patients in proteomic study might be compromised due to their low expression in serum. Therefore, to clarify the phenotypes and molecular mechanisms of new-onset metabolic complications of COVID-19, it is important to precisely determine the alterations of lipids and metabolic proteins in patients without pre-existing metabolic diseases.

In this study, we retrospectively studied a cohort without pre-existing metabolic diseases, and found that elevated blood glucose and new-onset insulin resistance were induced in COVID-19 patients. Furthermore, we found that the secreted factors, myeloperoxidase was upregulated whereas apelin and myostatin were downregulated upon SARS-CoV-2 infection, which was potentially linked to the onset of insulin resistance. Mechanistically, virus infection elevated the expression of RE1-silencing transcription factor (REST), which transcriptionally regulated the above three metabolic factors to modulate glucose and lipid metabolism in COVID-19. Moreover, with the lipidomic study and short-chain fatty acid analysis, (±)5-HETE, (±)12-HETE, propionic acid, and isobutyric acid were identified as the potential biomarkers of COVID-19-induced metabolic dysregulation. Our study reported insulin resistance, instead of insulin deficiency, as the main pathophysiology behind the hyperglycemia observed in COVID-19 patients, and further illustrated the underlying mechanism, which will be of great significance in clinical treatment and follow-up study for metabolic complications of COVID-19 patients.

## Results

### Glucose and lipid metabolic dysregulation in sera of COVID-19 patients

**Fig. 1:**
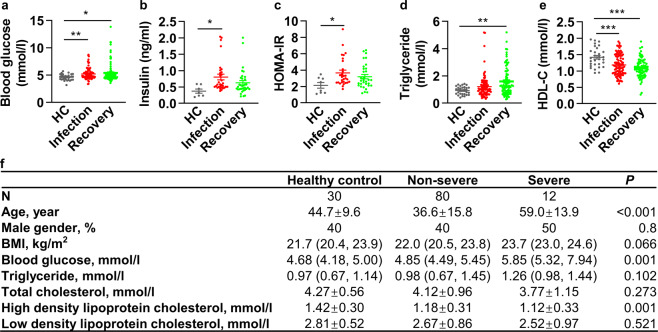
Glucose and lipid metabolic dysregulation in sera of COVID-19 patients. **a** Fasting blood glucose in the healthy control group and COVID-19 infection and recovery group (HC, healthy control, *n* = 30; COVID-19 infection and recovery, *n* = 92). **b** Fasting blood insulin in the healthy control group and COVID-19 group (HC, *n* = 10; COVID-19 infection and recovery, *n* = 34). **c** Homeostatic model assessment for insulin resistance (HOMA-IR) in the healthy control group and COVID-19 group (HC, *n* = 10; COVID-19 infection and recovery, *n* = 34). **d** Blood triglyceride in the healthy control group and COVID-19 group (HC, *n* = 30; COVID-19 infection and recovery, *n* = 92). **e** Blood high-density lipoprotein–cholesterol (HDL-C) in the healthy control group and COVID-19 group (HC, *n* = 30; COVID-19 infection and recovery, *n* = 92). **f** Basic characteristics and metabolic parameters of the individuals in healthy control, non-severe, and severe groups are shown. Error bars represent SEM. *P < 0.05; **P < 0.01; ***P < 0.001. See also Supplementary Fig. S1

In order to investigate the metabolic dysregulation in COVID-19 patients, the clinical data of patients diagnosed with COVID-19 between January 22, 2020 and April 7, 2020 in Guangzhou Eighth People’s Hospital, were retrospectively collected. A total of 124 COVID-19 patients (32 with and 92 without metabolic-related diseases) and 30 cases of healthy controls were further studied. First, the clinical measurements of 92 COVID-19 patients without pre-existing metabolic-related diseases were analyzed in comparison to 30 healthy controls. Strikingly, we found that the blood glucose, insulin, homeostatic model assessment for insulin resistance (HOMA-IR), as well as triglyceride were elevated upon SARS-CoV-2 infection in comparison to healthy control (Fig. 1a–d), whereas the HDL-C was significantly reduced (Fig. 1e). Total cholesterol and low-density lipoprotein–cholesterol (LDL-C) were modestly reduced (Supplementary Fig. 1a, b). Importantly, the alterations of these metabolic parameters sustained in the recovery phase (Fig. 1a–e), indicating a long-term impact of virus infection on systemic metabolism. As shown in Fig. 1f, for these COVID-19 individuals, 80 were non-severe patients and 12 were severe ones. The mean age was 44.7 ± 9.6 years in the healthy control group, 36.6 ± 15.8 years in non-severe group, and 59.0 ± 13.9 years in the severe group (*P* < 0.001). There were no significant gender differences among these three groups. The Body mass index (BMI) was 21.7 (20.4, 23.9) in healthy control group, 22.0 (20.5, 23.8) in non-severe group, and 23.7 (23.0, 24.6) in severe group (*P* = 0.066). We next compared these metabolic parameters among the non-severe, severe patients and healthy control. The elevated blood glucose, as well as reduced HDL-C were also observed in non-severe and severe COVID-19 patients compared with healthy control (Fig. 1f). Taken together, our data suggest that SARS-CoV-2 infection increases blood glucose and insulin levels, resulting in new-onset insulin resistance. Furthermore, virus infection reduces HDL-C level.

We next compared the clinical measurements between these two groups: patients with or without pre-existing metabolic-related diseases. Notably, much higher proportion of severe subjects was observed in patients with pre-existing metabolic-related diseases (37.5% vs. 13.0%, *P* = 0.006), as well as elevated C-reactive protein (18.04 (10.00, 37.02) vs. <10.00, *P* < 0.001), lactate dehydrogenase (222.96 ± 77.53 vs. 182.35 ± 59.11, *P* = 0.010), procalcitonin (0.0629 (0.0372, 0.1375) vs. 0.0399 (0.0316, 0.0620), *P* = 0.018), alanine aminotransferase (23.00 (16.90, 34.30) vs. 18.90 (14.75, 27.25), *P* = 0.034), creatinine (71.60 (59.80, 84.20) vs. 59.40 (52.65, 75.30), *P* = 0.021) and decreased estimated glomerular filtration rate (125.0 ± 38.7 vs. 145.1 ± 38.7, *P* = 0.009) (Supplementary Fig. 1c), indicating that pre-existing metabolic-related diseases exacerbate COVID-19 progress, which is consistent with previous studies.^1–3,27^

### Lipidomics profiling in sera of COVID-19 patients

**Fig. 2:**
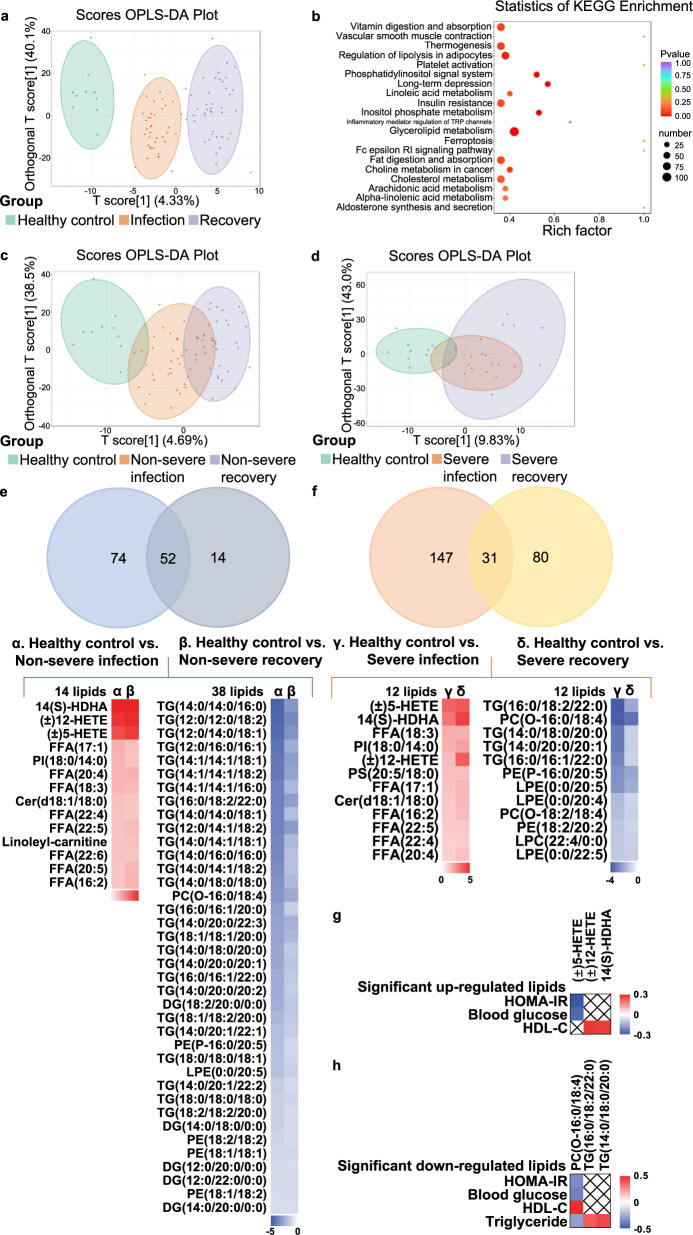
Lipidomics profiling in sera of COVID-19 patients. **a** Scores orthogonal projection to latent structure discriminant analysis (OPLS-DA) plot in healthy control, infection, and recovery group are shown. **b** Kyoto Encyclopedia of Genes and Genomes (KEGG) pathway enrichment of differential lipids in healthy control, infection, and recovery group are shown. **c** Scores OPLS-DA plot in healthy control, non-severe infection, and non-severe recovery group are shown. **d** Scores OPLS-DA plot in healthy control, severe infection, and severe recovery group are shown. **e** Upper panel, Venn diagram of differential lipids between two comparisons: (1) differential lipids in non-severe infection vs. healthy control, (2) non-severe recovery vs. healthy control. Lower panel, the heatmap is shown to represent the alterations of differential lipids in non-severe infection phase and recovery phase against healthy control, respectively. Data are shown as log_2_ fold change (left, significantly upregulated lipids were shown in red; right, significantly downregulated lipids in blue). **f** Upper panel, Venn diagram of differential lipids between two comparisons: (1) differential lipids in severe infection vs. healthy control, (2) severe recovery vs. healthy control. Lower panel, the heatmap is shown to represent the alterations of differential lipids in the severe infection phase and recovery phase against healthy control, respectively. Data are shown as log_2_ fold change (left, significantly upregulated lipids were shown in red. Right, significantly downregulated lipids in blue). **g** The heatmap is shown to represent the correlation between these significantly upregulated lipids and metabolic parameters. Data are shown as R-square. **h** The heatmap is shown to represent the correlation between these significantly downregulated lipids and metabolic parameters. Data are shown as R-square. See also Supplementary Fig. S2

Given the significant alterations in lipids as described above, we next analyzed the lipid components in sera of COVID-19 patients by using LC-MS system. The sera from 40 individuals of infection phase and recovery phase, and 10 from healthy control were analyzed, and in total 631 lipids were identified with authentic compound library searching. Scores orthogonal projection to latent structure discriminant analysis (scores OPLS-DA) plot showed significant differences among healthy control, infection and recovery group, suggesting that COVID-19 significantly altered serum lipids (Fig. 2a). Based on differential lipids among three groups, the KEGG enrichment study showed that these lipids were significantly enriched among the pathways of insulin resistance and cholesterol metabolism (Fig. 2b). In subgroup studies, as shown in Fig. 2c, d and Supplementary Fig. 2a–d, significant changes were observed in the infection phase and recovery phase of both non-severe and severe patients in comparison to the health group. We next searched for the key lipids which were altered upon virus infection and sustained even after virus elimination. We identified 52 significantly altered lipids which overlapped between non-severe infection vs. healthy control and non-severe recovery vs. healthy control (Fig. 2e). Similarly, 31 significantly altered lipids were found in both the infection phase and recovery phase in the severe group (Fig. 2f). To further analyze these lipids, we found that 14 lipids were upregulated in the non-severe group in both infection and recovery phase, and 38 lipids were downregulated (Fig. 2e). Moreover, in the severe group, 12 lipids were upregulated in both infection and recovery phases, and 12 lipids were downregulated (Fig. 2f). Notably, in the severe group, another seven lipids were downregulated in the infection phase but upregulated in the recovery phase in comparison to the healthy control group (Supplementary Fig. 2e).

Next, we conducted correlation analyses between these significantly altered lipids and metabolic parameters. (±)5-HETE, (±)12-HETE, and 14(S)-HDHA were most robustly upregulated in both infection and recovery phases of non-severe and severe patients. Among these three lipids, (±)5-HETE was negatively correlated with HOMA-IR (*R*^2^ = −0.315, *P* = 0.075) and blood glucose (*R*^2^ = −0.268, *P* = 0.125). (±)12-HETE and 14(S)-HDHA showed positive correlation with HDL-C (*R*^2^ = 0.275, *P* = 0.115; *R*^2^ = 0.267, *P* = 0.126) (Fig. 2g). Next, for lipids with most robust reduction in infection and recovery phase of non-severe and severe patients, PC (O-16:0/18:4) showed a negative correlation with HOMA-IR (*R*^2^ = −0.339, *P* = 0.054), blood glucose (*R*^2^ = −0.365, *P* = 0.034) and triglyceride (*R*^2^ = −0.285, *P* = 0.103), but a positive correlation with HDL-C (*R*^2^ = 0.478, *P* = 0.004). TG (16:0/18:2/22:0) and TG (14:0/18:0/20:0) showed a positive correlation with triglyceride (*R*^2^ = 0.368, *P* = 0.032; *R*^2^ = 0.414, *P* = 0.015) (Fig. 2h). Furthermore, correlation analyses between all the 631 lipids and metabolic parameters were also performed (Supplementary Fig. 2f–i and Supplementary Table 2). Several lipids, such as TG (14:0/20:4/22:0) (correlated with HOMA-IR, *R*^2^ = 0.553, *P* = 0.01), FFA (18:4) (with HOMA-IR, *R*^2^ = −0.467, *P* = 0.006), PE(18:0/22:6) (with blood glucose, *R*^2^ = 0.446, *P* = 0.008), PS (20:3/18:0) (with blood glucose, *R*^2^ = −0.599, *P* < 0.001), PC (O-16:2/18:1) (with HDL, *R*^2^ = 0.676, *P* < 0.001), Cer (d18:1/17:0) (with HDL, *R*^2^ = −0.478, *P* = 0.004), TG (18:1/18:1/18:2) (with triglyceride, *R*^2^ = 0.652, *P* < 0.001), and PS (18:1/20:0) (with triglyceride, *R*^2^ = −0.420, *P* = 0.013), showed significant correlation with metabolic parameters that could potentially serve as biomarkers of COVID-19.

### The short-chain fatty acids profiling in sera of COVID-19 patients

**Fig. 3:**
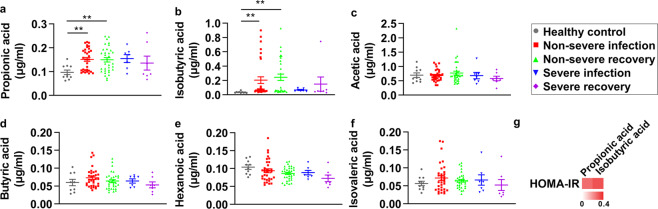
The short-chain fatty acids profiling in sera of COVID-19 patients. **a**–**f** Sera short-chain fatty acids concentration in healthy control, non-severe infection, non-severe recovery, severe infection, and severe recovery group (healthy control group, *n* = 10, non-severe infection and recovery group, *n* = 33, severe infection and recovery group, *n* = 7). **g** The heatmap is shown to represent the correlation between propionic acid and isobutyric acid and metabolic parameters. Data are shown as R-square. Error bars represent SEM. **P < 0.01. See also Supplementary Fig. S3

As short-chain fatty acids (SCFAs) analysis was still not reported in COVID-19 patients sera, to further explore potential factors involved in COVID-19-related metabolic dysregulation, GC–MS analysis was applied for SCFAs profiling in the same cohort of lipidomic study (Supplementary Fig. 3a). Acetic acid, propionic acid, isobutyric acid, butyric acid, isovaleric acid, and hexanoic acid were quantified in our study, whereas valeric acid was not detected due to its extremely low concentration in sera. Interestingly, propionic acid and isobutyric acid were significantly upregulated in non-severe COVID-19 patients in both infection and recovery phases, and trended up in severe patients in both phases (Fig. 3a, b). However, the other four SCFAs were not significantly altered (Fig. 3c–f).

For correlation studies, we examined propionic acid and isobutyric acid with HOMA-IR. As shown in Fig. 3g, propionic acid and isobutyric acid showed a positive correlation with HOMA-IR (*R*^2^ = 0.266, *P* = 0.135; *R*^2^ = 0.314, *P* = 0.075).

### The measurements of secreted metabolic factors in sera of COVID-19 patients

**Fig. 4:**
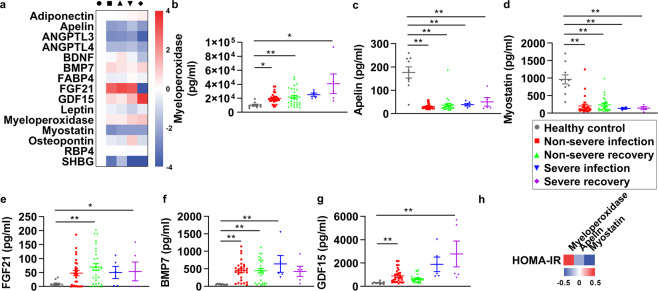
The measurements of secreted metabolic factors in sera of COVID-19 patients. **a** The heatmap is shown to represent the alteration of secreted metabolic factors in non-severe infection, non-severe recovery group, severe infection, and severe recovery group against the healthy control group. Data are shown as log_2_ fold change, values of metabolic factors in the healthy control group were set as 0. Sera myeloperoxidase (**b**), apelin (**c**), myostatin (**d**), fibroblast growth factor 21 (FGF21) (**e**), bone morphogenetic protein 7 (BMP7) (**f**), and growth differentiation factor 15 (GDF15) (**g**) in healthy control (*n* = 10), non-severe infection (n = 29), non-severe recovery group (*n* = 29), severe infection (*n* = 5), and severe recovery group (*n* = 5) are shown. **h** The heatmap is shown to represent the correlation between myeloperoxidase, apelin, and myostatin and metabolic parameters. Data are shown as R-square. Error bars represent SEM. *P < 0.05; **P < 0.01. See also Supplementary Fig. S4

SARS-CoV-2 infection induced various alterations in the serum proteome of COVID-19 patients,^22–25^ which may have a further impact on the metabolic regulation in other tissues. However, all the serum proteome data were obtained by high throughput mass spectrometry analysis without further experimental validation. Given that most metabolic factors were of small molecular size and low biological concentration, they might be missed during mass spectra flow, and further misinterpreted by the data processing with inaccurate concentration.^28^ Therefore, to further understand the alterations of secreted metabolic factors, we selected the common and substantial metabolic factors involved in glucose and lipid metabolism (*n* = 15), and analyzed these factors in the sera of COVID-19 patients by Luminex assay. The fold changes of these factors in sera of non-severe or severe patients in infection and recovery phases compared with healthy control were shown as a heatmap in Fig. 4a. The metabolic factors with significant alterations were further shown in Fig. 4b–g. In detail, myeloperoxidase (MPO) was significantly upregulated in both non-severe and severe groups, which persisted in the recovery phase (Fig. 4b). In contrast, apelin and myostatin were downregulated and sustained in the recovery phase (Fig. 4c, d). Other metabolic factors, including FGF21, BMP7, and GDF15, trended up upon virus infection (Fig. 4e–g), presumably due to a compensatory effect as these three secreted proteins were involved in positively maintaining metabolic homeostasis.

Considering that MPO, apelin, and myostatin showed consistent alteration patterns upon COVID-19, which is in line with their previously identified regulatory roles, we next explored the potential association between MPO, apelin, and myostatin and metabolic parameters. We found that MPO showed a positive correlation with HOMA-IR (*R*^2^ = 0.441, *P* = 0.010), whereas apelin and myostatin showed a negative correlation with HOMA-IR (*R*^2^ = −0.256, *P* = 0.151; *R*^2^ = −0.497, *P* = 0.003) (Fig. 4h). The other ten metabolic factors without significant changes in COVID-19 patients are listed in Supplementary Fig. 4a–i. Notably, we found out despite that FABP4 showed no significant change in COVID-19 patients, its level was negatively correlated with HDL-C in patients with HDL ≤ 1.0 mmol/l (*R*^2^ = −0.403, *P* = 0.087) and positively correlated with triglyceride in patients with TG ≥ 1.7 mmol/l (*R*^2^ = 0.821, *P* = 0.089), suggesting that it may influence lipid metabolism (Supplementary Fig. 4j).

### The metabolic regulation of MPO, apelin, and myostatin in vitro

**Fig. 5:**
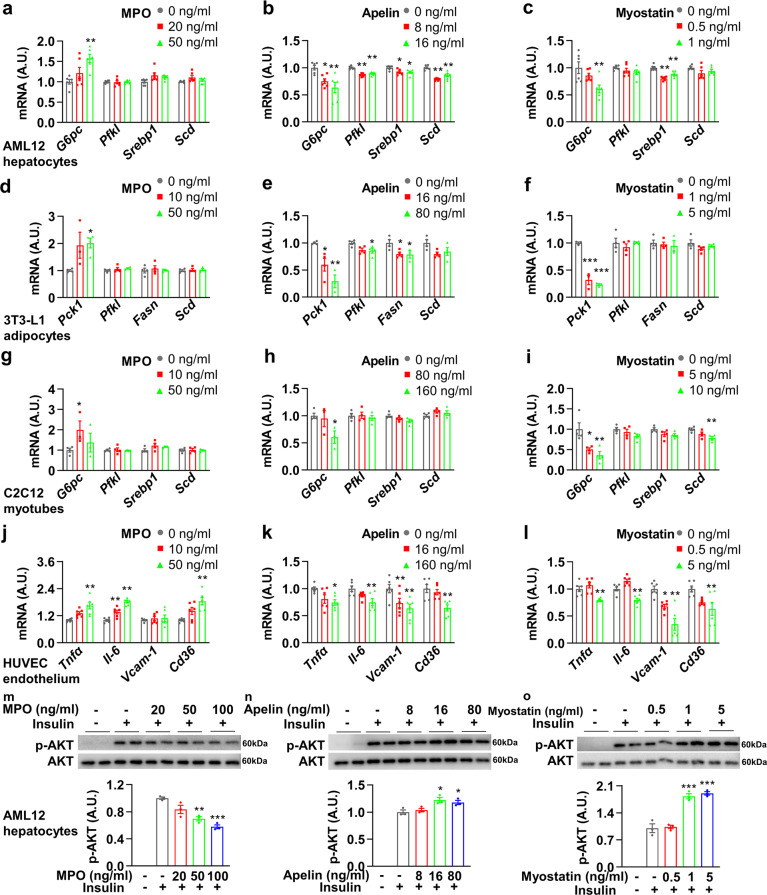
The metabolic regulation of myeloperoxidase, apelin, and myostatin in vitro. **a**–**c** AML12 cells were treated with myeloperoxidase (MPO), apelin, or myostatin for 24 h and subjected to real-time PCR (*n* = 6). **d**–**f** 3T3-L1 adipocytes were treated with myeloperoxidase (MPO), apelin, or myostatin for 24 h and subjected to real-time PCR (*n* = 3–4). **g**–**i** C2C12 myotubes were treated with MPO, apelin, or myostatin for 24 h and subjected to real-time PCR (*n* = 3–4). **j**–**l** HUVEC were treated with myeloperoxidase, apelin, or myostatin for 24 h and subjected to real-time PCR (*n* = 6). **m**–**o** AML12 cells were treated with MPO, apelin, or myostatin for 24 h following starvation with DMEM medium for 2 h, then 100 nM insulin was administrated for 15 min and subjected to western blot (*n* = 3, quantifications were shown below the blots). Error bars represent SEM. *P < 0.05; **P < 0.01; ***P < 0.001. See also Supplementary Fig. S5

We further examined the regulatory role of these three metabolic factors in metabolism in vitro. We first determined the effects in murine liver cell line AML12. The expression of gluconeogenesis gene *G6pc* was significantly upregulated after MPO treatment in a dose-dependent manner, whereas the glycogenolysis gene *Pfkl*, lipogenesis gene *Srebp1* and *Scd* were not altered (Fig. 5a). Furthermore, *G6pc* was significantly downregulated after apelin administration, as well as a modest reduction of *Pfkl*, *Srebp1*, and *Scd* (Fig. 5b). For myostatin treatment, *G6pc* and *Srebp1* were downregulated, whereas *Pfk1* and *Scd* were not altered (Fig. 5c). Likewise, these three factors were administrated in 3T3-L1 adipocytes and C2C12 myotubes. In adipocytes, the expression of gluconeogenesis gene *Pck1* was significantly upregulated after MPO treatment, whereas *Pfkl*, *Fasn*, and *Scd* were not altered (Fig. 5d). *Pck1* and *Pfkl* were significantly downregulated after apelin administration, with a modest reduction of *Fasn* (Fig. 5e). With myostatin treatment, *Pck1* was significantly downregulated, whereas *Pfkl*, *Fasn*, and *Scd* were not altered (Fig. 5f). In myotubes, MPO treatment elevated the expression of *G6pc* (Fig. 5g), apelin reduced the expression of *G6pc* (Fig. 5h), while myostatin decreased the expressions of *G6pc* and *Scd* (Fig. 5i). We further applied these three metabolic factors in human umbilical vein endothelial cells (HUVEC). Interestingly, MPO treatment increased the gene expressions of *Tnfα*, *Il-6*, and *Cd36*, suggesting an enhanced inflammation and cholesterol uptake effect (Fig. 5j). However, apelin or myostatin treatment decreased gene expression of *Tnfα*, *Il-6*, *Vcam-1*, and *Cd36* (Fig. 5k, l).

Lastly, the effects of these three factors on the insulin signaling pathway were studied. In AML12 cells, as shown in Fig. 5m–o, MPO illustrated a significant effect on reduction of phosphorylated-AKT upon insulin treatment, indicating induced insulin resistance. On contrary, apelin and myostatin treatment exhibited a significant increase of phosphorylated-AKT, indicating increased insulin sensitivity. Furthermore, we also conducted these experiments in 3T3-L1 adipocytes, 3T3-L1 pre-adipocytes, C2C12 myotubes, and C2C12 myoblasts (Supplementary Fig. 5a–j), and we found that MPO could induce insulin resistance in adipocytes, pre-adipocytes, and myotubes while apelin and myostatin exhibited a more specific effect on enhancing insulin sensitivity in adipocytes and pre-adipocytes.

### MPO, apelin, and myostatin were regulated by transcription factor REST

**Fig. 6:**
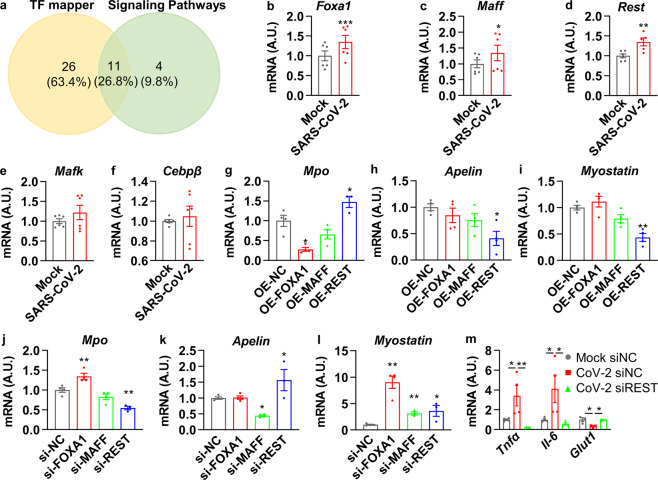
MPO, apelin, and myostatin were regulated by transcription factor REST. **a** Venn diagram of potential transcription factors that could bind to the promoter region of MPO, apelin, and myostatin from two online ChIP-seq datasets: TF mapper and Signaling Pathways. **b**–**f** HUVEC were infected with SARS-CoV-2 (MOI = 0.005) for 18 h and subjected to real-time PCR (*n* = 6). **g**–**i** HUVEC were transfected with FOXA1, MAFF, or REST plasmid for overexpression for 72 h and subjected to real-time PCR (*n* = 4). **j**–**l** HUVEC were infected with 10 nM control siRNA (si-NC), FOXA1 siRNA (si-FOXA1), MAFF siRNA (si-MAFF), REST siRNA (si-REST) for 72 h and subjected to real-time PCR (*n* = 4). **m** HUVEC were infected with 10 nM control siRNA (si-NC), REST siRNA (si-REST) for 48 h, then infected with SARS-CoV-2 (MOI = 0.005) for 24 h and subjected to real-time PCR (*n* = 4). Error bars represent SEM. *P < 0.05; **P < 0.01; ***P < 0.001. See also Supplementary Fig. S6

To further illustrate the mechanisms regarding how MPO, apelin, and myostatin were regulated upon SARS-CoV-2 infection, we first aimed to identify the transcription factors involved in the regulation of these three metabolic factors. To address this, we applied bioinformatic study with two online chromatin immunoprecipitation sequencing (ChIP-seq) datasets, TF mapper and Signaling Pathways. By using these two methods, a total of 41 transcription factors were identified with putative binding to the promoter region of MPO, apelin, and myostatin, whereas 11 transcription factors were found to be overlapped in both datasets (Fig. 6a). The gene expression of these 11 transcription factors after SARS-CoV-2 infection, reported from the previous transcriptome studies,^29^ is illustrated in Supplementary Fig. 6a. The top five upregulated proteins, including MAFF, MAFK, REST, CEBPβ, and FOXA1, were selected for further validation. Interestingly, after SARS-CoV-2 infection, significantly upregulated mRNAs of *Foxa1*, *Maff*, and *Rest* were observed, whereas no significant alterations of *Mafk* and *Cebpβ* mRNA were noticed (Fig. 6b–f). Hence, FOXA1, MAFF, and REST were further investigated with gain/loss of function studies.

Importantly, overexpression of REST showed significant upregulation of *Mpo* mRNA and reduction of *Apelin* and *Myostatin* mRNA; overexpression of FOXA1 showed significant downregulation of *Mpo* mRNA, but no change for *Apelin* or *Myostatin* mRNA; overexpression of MAFF failed to alter mRNA of *Mpo*, *Apelin*, and *Myostatin* (Fig. 6g–i). Vice Versa, knockdown of REST showed a significant reduction of *Mpo* mRNA and increase of *Apelin* and *Myostatin* mRNA; knockdown of FOXA1 showed a significant increase of *Mpo* and *Myostatin* mRNA, but no change for *Apelin* mRNA; knockdown of MAFF showed a significant reduction of *Apelin* and *Myostatin* mRNA, but no change for *Mpo* mRNA (Fig. 6j–l). These data suggest that REST transcriptionally regulates gene expressions of *Mpo*, *Apelin*, and *Myostatin* upon SARS-CoV-2 infection. Therefore, the expression of REST upon virus infection was manipulated for further validation. We found that infection of SARS-CoV-2 could elevate the expression of *Tnfα* and *Il-6*, and downregulate the expression of *Glut1*, and these effects were blocked by the knockdown of REST (Fig. 6m). Taken together, these data suggest that COVID-19-associated metabolic abnormalities are at least partially dependent on the transcription factor REST and its downstream genes, which include MPO, apelin, and myostatin.

## Discussion

Recent studies have reported that COVID-19 patients with pre-existing metabolic-related diseases suffered from a worse prognosis,^1–4,27^ yet very few cases were reported concerning the deterioration of glucose and lipid metabolism in patients without pre-existing metabolic-related diseases.^9,12,18^ In this study, we observed the new-onset insulin resistance, elevated blood glucose, as well as reduced HDL-C in COVID-19 patients, which persisted even after virus elimination. Mechanistically, we found that SARS-CoV-2 infection induced the expression of REST, which transcriptionally modulated the gene expression of MPO, apelin, and myostatin, resulting in glucose and lipid metabolic dysregulation. Moreover, our data revealed that (±)5-HETE, (±)12-HETE, propionic acid, and isobutyric acid could be potential biomarkers for COVID-19-associated metabolic dysregulation.

Despite a few clinical reports observed the increased blood glucose in COVID-19 patients,^7–10^ the underlying mechanisms remain unclear. Previously it was widely assumed that pancreatitis with deficiency of insulin secretion was the potential cause of hyperglycemia upon virus infection.^15,16^ This hypothesis was supported by elevated pancreatic enzymes in up to 31% of COVID-19 patients,^30,31^ necrosis and hemorrhage findings in the pancreas in postmortem study,^16^ and case report of autoantibody-negative type 1 diabetes after SARS-CoV-2 infection.^17^ However, very recently, Montefusco et al. observed hyperinsulinemia in 10 COVID-19 patients,^18^ and a higher prevalence of elevated C-peptide was also reported in acute respiratory distress syndrome (ARDS) patients with COVID-19 in comparison to ARDS patients without COVID-19.^19^ Importantly, our data indicated that new-onset of insulin resistance, instead of insulin deficiency, was the potential mechanism resulting in hyperglycemia upon SARS-CoV-2 infection. More importantly, this insulin resistance condition would sustain even after viral elimination, implying a potential long-term pathology in COVID-19 patients. These findings warrant more surveillance and even treatment during follow-up for the patients with COVID-19-induced metabolic dysregulations.

Second, we found that SARS-CoV-2 infection could modulate the expression of MPO, apelin, and myostatin by up-regulating transcription factor REST, which may lead to glucose and lipid metabolic disorders. MPO is a heme-containing enzyme that reacts with hydrogen peroxide to generate hypochlorite and other halide oxidants. Released by neutrophils during infection, MPO facilitates the formation of neutrophil extracellular traps (NET) as an immunity reaction.^32^ Interestingly, downregulation of MPO in mice was reported to improve insulin resistance.^33,34^ Apelin is an endogenous ligand of the G protein-coupled receptor APJ (apelin junction receptor), which was reported to inhibit angiotensin-converting enzyme (ACE) and reduce angiotensin II production.^35^ Recent reports illustrated that administration of apelin would improve insulin sensitivity in overweight men.^36^ Myostatin was originally found as a regulator of muscle mass, whereas recent works have shown controversial effects of myostatin on insulin resistance. Zhang et al. reported that mice with whole-body knockout of myostatin exhibited improved insulin resistance,^37^ whereas specific overexpression of myostatin in murine fat tissue would increase insulin sensitivity,^38^ indicating complex organ-specificity of myostatin. Consistent with and beyond these previous findings, our data suggest that MPO was positively correlated with HOMA-IR, promoting gene expression of gluconeogenesis and inflammation, whereas apelin and myostatin exerted opposite effects. Our current study clarified these three metabolic factors as a novel signature pattern in the regulation of COVID-19-induced insulin resistance.

We further identified the transcription factor REST as the master modulator of MPO, apelin, and myostatin upon SARS-CoV-2 infection. REST was originally described as a silencer of neuronal genes outside the central nervous system and its expression was restricted to non-neuronal cells and undifferentiated neural progenitors.^39^ Recent works illustrated its role out of the brain, as mice with overexpression of REST in the pancreas showed impaired glucose tolerance.^40^ Altogether, our findings showed a potential mechanism behind COVID-19-induced insulin resistance, suggesting MPO, apelin, myostatin, as well as the transcription factor REST as potential manipulated targets for the treatment of metabolic dysregulation in COVID-19 patients.

Last, we also identified several lipids that were correlated with glucose and lipid dysregulation, which could be applied as potential biomarkers in the diagnosis and follow-up of COVID-19 study. For lipidomic study, (±)5-HETE and (±)12-HETE were significantly upregulated after infection, in consistence with a previous finding,^21^ and our data showed that (±)5-HETE were negatively correlated with HOMA-IR and blood glucose, and (±)12-HETE were positively correlated with HDL-C, suggesting a potential favorable role. As the first sera short-chain fatty acids study in COVID-19 patients so far, we found that propionic acid and isobutyric acid were significantly upregulated after infection. Propionic acid was reported to activate sympathetic nervous system in mice to induce insulin resistance and hyperinsulinemia in human,^41^ whereas isobutyric acid showed benefits on glucose and lipid metabolism in vitro.^42^ In our data, propionic acid and isobutyric acid were positively correlated with HOMA-IR, suggesting a potential deteriorative role. In conclusion, these lipids could be used as potential biomarkers to reflect metabolic dysregulation of COVID-19 patients.

In summary, our current study indicates the new-onset and persisting insulin resistance in COVID-19 patients, and elucidates the potential underlying mechanisms that involved metabolic factors including MPO, apelin, and myostatin, which are transcriptionally regulated by REST upon SARS-CoV-2 infection. Therefore, this study extends our understanding of the extrapulmonary manifestation of COVID-19 in metabolic complications, urging more intensive attention in the treatment for these COVID-19-induced metabolic defects upon admission and during follow-up.

### Limitations of the study

Our current study has several limitations. First, as all the patients and data were from a single center, potential bias was inevitable. Second, though we identified a potential mechanism of REST-metabolic factors axis in the regulation of metabolic dysfunctions upon SARS-CoV-2 infection, an in vivo manipulation of these potential targets should be conducted with SARS-CoV-2 to further verify their effects on metabolic dysregulation.

## Materials and methods

### Reagents

Chemicals for lipidomic analysis, including methanol (Cat. 106007), acetonitrile (Cat. 113358), isopropanol (Cat. 101040), formic acid (98%) (Cat. 00940), and isopropanol (Cat. 101040) were purchased from Merck, Germany. Ammonium formate (Cat. A11550) and dichloromethane (Cat. AC6100500) were purchased from Fisher Chemicals, USA. Standard substance of 12:0 Lyso PC (Cat. 855475), Cer(d18:1/4:0) (Cat. 860524), PC(13:0/13:0) (Cat. 850340), DG(12:0/12:0) (Cat. 800812) and TG(17:0/17:0/17:0) (Cat. 860903) were purchased from Avanti Polar Lipids, USA. Chemicals for short-chain fatty acid analysis were purchased from CNW Technologies, Germany, including acetic acid (Cat. CAEQ-4-011245-0500), propionic acid (Cat. CFEQ-4-510510-0100), isobutyric acid (Cat. CFEQ-4-480113-0005), butyric acid (Cat. CFEQ-4-480113-0005), isovaleric acid (Cat. CFLD-I108280), valeric acid (Cat. CFEQ-4-534013-0005), hexanoic acid (Cat. CFEQ-4-470115-0100), 2-methylpentanoic acid (Cat. CFAH-8-18774-0100) and methyl tertiary butyl ether (Cat. CDCT-C15084600). Luminex assays were purchased from R&D and Millipore as Luminex Human Magnetic Assay kit (LXSAHM-13) and MILLIPLEX MAP Human Myokine Magnetic Bead Panel (HMYOMAG-56K). Proteins of human Myeloperoxidase (Cat. 11917-H08B), human Apelin (Cat. HY-P1944), and human Myostatin (Cat. 50441-M01H) were purchased from Sino Biological Company, China. RNA extraction reagent (AG21102), Evo M-MLV Reverse Transcription kit (AG11711), SYBR Green Premix Pro Taq HS qPCR kit (AG11701) were purchased from Accurate Biotechnology Co., Ltd, China. The primary antibody of Phospho-Akt (Ser473) (D9E), Akt (pan) (C67E7), and the secondary antibody of Anti-rabbit IgG, HRP-linked Antibody (7074) were purchased from Cell Signaling Technology, USA. The recombinant DNA pcDNA 3×FLAG-FOXA1, pcDNA 3×FLAG-MAFF, and pcDNA 3×FLAG-REST were purchased from Guangzhou DZ Technologies, China.

### Study design and participants

This retrospective cohort study was approved by the Institutional Review Board of Guangzhou Eighth People’s Hospital, with written informed consent collected.

The data of patients diagnosed with COVID-19 between January 22, 2020 and April 7, 2020 were retrospectively collected. The exclusion criteria were: (1) asymptomatic patients, (2) incomplete medical records (transferring to other designated hospitals), and (3) patients refused to be recruited in this study. For healthy control, 30 healthy volunteers with matched age and gender distribution were included. A total of 124 COVID-19 patients (32 with and 92 without metabolic-related diseases) and 30 cases for healthy control were further studied and analyzed.

### Data and blood sample collection

Data of demographics, clinical features, laboratory and imaging results and outcomes were retrieved with a standardized data collection form by two independent physicians and were further analyzed by an integrated research team of physicians and statisticians.

Blood samples were collected from 40 patients without pre-existing metabolic-related diseases, including hypertension, dyslipidemia, diabetes mellitus, and nonalcoholic fatty liver disease, in the cohort of COVID-19. Consecutive samples in the phase of infection and recovery were collected. Of note, the blood samples of the infection phase were collected on the second day of admission with overnight fasting, while the blood samples of the recovery phase were collected within 3 days before discharge after overnight fasting. For comparison, the blood samples of ten healthy volunteers were collected as well. All the blood samples were disinfected at the temperature of 56 °C for 30 min.

### Definition

COVID-19 was diagnosed according to the Clinical Guideline for COVID-19 Diagnosis and Treatment published by the National Health Commission of China (Trial Version 7). The infection phase was defined as the period during patients’ hospitalization with positive qPCR test for SARS-CoV-2 and COVID-19 relevant symptoms, whereas the recovery phase was defined as the period when elimination of virus was confirmed by two negative qPCR tests for SARS-CoV-2 in two consecutive days shortly before discharge. Type 2 diabetes (T2D) status was defined based on patients’ medical history and guideline for the prevention and control of T2D in China^43^ as fasting glucose ≥7.0 mmol/l or 2 h OGTT blood glucose ≥11.1 mmol/l. Hypertension was diagnosed when systolic blood pressure ≥140 mm Hg and/or diastolic blood pressure ≥90 mm Hg.^44^ Dyslipidemia was diagnosed with patients’ medical history and guidelines for the management of dyslipidemia in adults.^45^ In detail, abnormal level of blood lipids was defined as either triglyceride (TG) ≥ 2.3 mmol/l, total cholesterol (TC) ≥ 6.2 mmol/l, high-density lipoprotein–cholesterol (HDL-C) ≤ 1.0 mmol/l or low-density lipoprotein–cholesterol (LDL-C) ≥ 4.1 mmol/l. Nonalcoholic fatty liver disease was diagnosed based on the guidelines of prevention and treatment for nonalcoholic fatty liver disease,^46^ with hepatic abnormalities (biochemical or with ultrasound) in the absence of significant alcohol consumption (>21 drinks per week for men and >14 drinks per week for women, for 2 years) and in the absence of other etiologies for hepatic steatosis or chronic liver disease.

### Short-chain fatty acid analysis

Samples of sera were thawed and vortexed for 1 min prior to analysis. 50 μl of one sample was added to a 1.5 ml EP tube and 100 μl of phosphoric acid (36% v/v) solution was added to the EP tube. The mixture was vortexed for 3 min. 150 μl methyl tert-butyl ether (MTBE, containing internal standard) solution was then added. The mixture was vortexed for 3 min and ultrasonicated for 5 min. Afterward, the mixture was centrifuged at 12000 r/min for 10 min at the temperature of 4 °C. The supernatant was collected and used for GC–MS/MS analysis.

An Agilent 7890B gas chromatograph coupled with a 7000D mass spectrometer with a DB-5MS column (30 m length × 0.25 mm i.d. × 0.25 μm film thickness, J&W Scientific, USA) was employed for GC–MS/MS analysis. Helium was used as the carrier gas at a flow rate of 1.2 ml/min. Injections were made in the splitless mode and the injection volume was 2 μl. The oven temperature was maintained at 90 °C for 1 min, ramped up to 100 °C at a rate of 25 °C /min, to 150 °C at a rate of 20 °C /min, holding on for 0.6 min, to 200 °C at a rate of 25 °C/min, holding on for 0.5 min and followed by a 3 min operation. All samples were analyzed in the multiple reaction monitoring mode. The temperatures of the injector inlet and transfer lines were 200 °C and 230 °C respectively. SCFAs contents were then detected using MetWare (http://www.metware.cn/) based on the Agilent 7890B-7000D GC–MS/MS platform.

### Lipidomic analysis

Samples were thawed on ice, whirled for around 10 s, and then centrifuged with 3000 rpm at 4 °C for 5 min. In total, 50 μl of each sample was homogenized with 1 ml of the mixture (including methanol, MTBE, and internal standard mixture), and was whirled for 15 min. In all, 200 μl of water was added to the mixture and whirled for 1 min. The mixture was then centrifuged at 12,000 rpm at 4 °C for 10 min. In total, 500 μl supernatant was extracted and concentrated. The concentrated powder was dissolved with 200 μl solvent B (acetonitrile/isopropanol (10/90 V/V, 0.1% formic acid, 10 mmol/l ammonium formate)) and was taken for LC-MS/MS analysis.

The extracts of samples were analyzed using an LC-ESI-MS/MS system (UPLC, ExionLC AD https://sciex.com.cn/; MS, QTRAP® System, https://sciex.com/). The analytical conditions were as follows: UPLC: column, Thermo Accucore™ C30 (2.6 μm, 2.1 mm × 100 mm). Solvent system, A: acetonitrile/water (V/V 60/40), 0.1% formic acid and 10 mmol/l ammonium formate; B: acetonitrile/isopropanol (V/V 10/90), 0.1% formic acid and 10 mmol/l ammonium formate. Gradient program was A/B (V/V 80:20) at 0 min, 70:30 at 2.0 min, 40:60 at 4 min, 15:85 at 9 min, 10:90 at 14 min, 5:95 at 15.5 min, 5:95 at 17.3 min, 80:20 at 17.3 min, 80:20 at 20 min, with flow rate 0.35 ml/min at 45 °C. The injection volume was 2 μl. The effluent was alternately connected to an ESI-triple quadrupole-linear ion trap (QTRAP)-MS.

Linear ion trap (LIT) and triple quadrupole (QQQ) scans were acquired with a triple quadrupole-linear ion trap mass spectrometer (QTRAP). The QTRAP^®^ LC-MS/MS System was equipped with an ESI Turbo Ion-Spray interface and controlled by Analyst 1.6.3 software (Sciex). The ESI source operation 35 parameters were as follows: ion source, turbo spray; source temperature, 500 °C; ion-spray voltage (IS), 5500 V (positive), −4500 V (negative); ion source gas 1 (GS1), gas 2 (GS2), and curtain gas (CUR) were set at 45, 55, and 35 psi, respectively; the collision gas (CAD) level was set at medium. Instrument tuning and mass calibration were performed with 10 and 100 μmol/l polypropylene glycol solutions in QQQ and LIT modes, respectively. QQQ scans were acquired from multiple reaction monitoring (MRM) experiments with collision gas (nitrogen) set to 5 psi. Declustering potential (DP) and collision energy (CE) for individual MRM transitions were conducted with further DP and CE optimization, respectively. A specific set of MRM transitions was monitored for each period according to the metabolites eluted within this period.

### The measurement of metabolic factors

A Luminex Human Magnetic Assay (LXSAHM-13, R&D) that measured adiponectin, angiopoietin-like protein 3 (ANGPTL3), angiopoietin-like protein 4 (ANGPTL4), brain-derived neurotrophic factor (BDNF), bone morphogenetic protein 7 (BMP7), fatty acid-binding protein 4 (FABP4), growth differentiation factor 15 (GDF15), insulin, myeloperoxidase, osteopontin, retinol-binding protein 4 (RBP4), sex hormone-binding globulin (SHBG) and Leptin were performed according to the manufacturers’ instructions. A MILLIPLEX MAP Human Myokine Magnetic Bead Panel Assay (HMYOMAG-56K, Millipore) that measured apelin, fibroblast growth factor 21 (FGF21), and mysostatin were also performed according to the manufacturers’ instructions. Briefly, the detection beads were incubated with standards or samples overnight at 4 °C. Then the mixture was washed and incubated with a biotinylated detection antibody. After incubation and washing, the beads were incubated with a complex of streptavidin with phycoerythrin. The intensities of fluorescent were developed on a Luminex 200 Analyzer (Luminex Corporation, USA). StatLia immunoassay software was used for data analysis (Brendan Technologies, USA). All samples were taken in two technical repeats.

### Cell culture

HUVEC were cultured in F-12K medium, supplemented with 10% fetal bovine serum (FBS), 0.1 mg/ml heparin, 30 µg/ml endothelial cell growth supplement, and 100 IU/ml penicillin and 100 µg/ml streptomycin. Mouse AML12 cells were cultured in DMEM/F12 medium supplemented with 10% FBS, and 100 IU/ml penicillin and 100 µg/ml streptomycin, a mixture of 1 mg/ml insulin, 0.55 mg/ml transferrin, 0.5 ug/ml selenium and 100 nM dexamethasone. 3T3-L1 pre-adipocytes and C2C12 cells were cultured in DMEM medium supplemented with 10% FBS and 100 IU/ml penicillin and 100 µg/ml streptomycin. All cells were seeded in a humidified atmosphere at 37 °C with 5% CO_2_. All culture reagents were purchased from Gibco. For differentiation, the 3T3-L1 pre-adipocytes were cultured in 12-well plate. After full confluence, cells were exposed to DMEM medium supplemented with 0.5 mmol/l IBMX, 1 μmol/l dexamethasone, 10 μg/ml insulin, and 10% FBS for 2 days, and then replaced with DMEM supplemented with 5 μg/ml insulin and 10% FBS for 2 days. Then the cells were cultured in DMEM medium with 10% FBS for 4 days to gain full differentiation.^47^ The C2C12 myoblasts were cultured in 12-well plates. After full confluence, cells were exposed to DMEM medium supplemented with 1% FBS for 5 days to gain differentiation.^48^ For metabolic factors treatment, cells were treated with myeloperoxidase, apelin, and myostatin for 24 h and then harvested. For insulin sensitivity assay, cells were treated with myeloperoxidase, apelin, and myostatin for 24 h following starvation with DMEM medium without FBS supplement for 1–2 h, and then harvested 15 min after insulin (10 nM in 3T3-L1 cells and C2C12 cells, 100 nM in AML12 cells) were added. For small interfering RNA (siRNA) treatment, cells were transfected with siRNAs using Lipofectamine 3000 reagent (Invitrogen, USA) according to the manufacturer’s instructions. For transient transfection, cells were at ~70% confluency, and were transfected with plasmids using Lipofectamine 3000 reagent (Invitrogen, USA) according to the manufacturer’s instructions.

### SARS-CoV-2 infection

The hCoV-19/CHN/SYSU-IHV/2020 strain of SARS-CoV-2 (from Prof. Hui Zhang lab, Institute of Human Virology, Key Laboratory of Tropical Disease Control of Ministry of Education, Zhongshan School of Medicine, Sun Yat-sen University) was used for all experiments. All the experiments of live virus were performed in a Biosafety Level 3 laboratory. SARS-CoV-2 stocks were passaged in Vero E6 cells (ATCC). SARS-CoV-2 infections of HUVEC were performed at a multiplicity of infection of 0.005 for 24 h.

### Plasmids and small interfering RNA

pcDNA 3 encoding 3×FLAG-FOXA1, 3×FLAG-CEBPB, 3×FLAG-MAFF, and 3×FLAG-REST were generated using human genomic DNA as a template and restriction enzymes BamHI and XhoI.

SiRNAs of FOXA1, CEBPB, MAFF, and REST were synthesized and purchased from RiboBio (Guangzhou, China). The sequences are listed in Supplementary Table S1.

### Real-time PCR analysis

Cells were lysed on ice with Trizol reagent (Accurate Biotechnology, China) to extract total RNA. Single-stranded cDNA for real-time PCR was synthesized with 1 μg of total RNA according to the manufacturer’s instructions (Accurate Biotechnology, China). The target cDNA was amplified by real-time PCR and the results were normalized to GAPDH. The value of the control group was set to 1. The sequences of the primers are listed in Supplementary Table 1.

### Western blot analysis

Cells were lysed in ice-cold RIPA lysis buffer with 1 mM PMSF and extracted. Total proteins were separated on 10% SDS-polyacrylamide gels and transferred onto PVDF membranes (Millipore, USA). Blots were blocked for 1 h in 5% milk and then incubated overnight with primary antibodies (p-AKT and AKT, 1:1000 dilution) at 4 °C. The HRP-conjugated secondary antibodies (1:10000 dilution) were applied for the blots at room temperature for 1 h and scanned with an enhanced chemiluminescence detection system (Amersham Imager 600, USA). Each blot represents at least three independent biological repeats. ImageJ was used for quantification of the gray intensity for blots.

### Bioinformatics

Genome-wide binding details of myeloperoxidase (MPO), apelin (APLN), and myostatin (MSTN) for human based on ChIP-Seq/DNase-Seq/ATAC-Seq were obtained from TF mapper^49^ which was accessed in January 2021. The parameters used to obtain the binding details were as follows: species, human (GRCh38); IP, trans-acting factors; biological source, all; search by gene (*MPO*, *APLN,* and *MSTN*); portion of the gene to query, all. Binding details were obtained separately for each gene and the results were downloaded in table format. Transcription factors with binding score ≥10.0 from each gene table were included in further overlap studies. A parallel search regarding transcription factors of myeloperoxidase, apelin, and myostatin was also conducted on the Signaling Pathways Project Ominer web tool^50^ in January 2021. The search criteria included Omics Category: Cistromics (Chip-Seq); Module Category, Transcription factors; Biosample Category, Human - all physiological systems. The search results were downloaded in table format. Top 50 transcription factors for each gene were overlapped to search for the mutual expression of the transcription factors. In all, 11 transcription factors were found in this process and further expression levels were searched from the transcriptome data in a COVID-19 study (access number: GSE147507).^29^

### Statistical analysis

For clinical data, continuous variables were expressed as mean ± standard deviation (SD) or median interquartile range (IQR), while categorical variables were expressed as frequencies and percentages. The analyses were performed using SPSS version 24.0 (IBM, Armonk, NY).

For omics analysis, the data were unit variance scaled, and then unsupervised principal component analysis (PCA) was performed using the statistics function prcomp within R (www.r-project.org). The data were log transform (log_2_) and mean centering was performed before OPLS-DA. VIP values were extracted from OPLS-DA result generated using R package MetaboAnalystR. Significantly regulated metabolites between groups were determined by VIP ≥ 1 and absolute Log_2_FC (fold change) ≥1. In order to avoid overfitting, a permutation test (200 permutations) was performed. Identified metabolites were further annotated and mapped with KEGG Compound database (http://www.kegg.jp/kegg/compound/) and KEGG Pathway database (http://www.kegg.jp/kegg/pathway.html), respectively. The pathways with significantly regulated metabolites mapped were then fed into metabolite sets enrichment analysis (MSEA), and their significance was determined by hypergeometric test *P* values. For correlation study between lipids and metabolic parameters, the statistics function correlation within R (www.r-project.org) were performed with Spearman’s Correlation.

For in vitro data, statistical data analysis was performed using Graphpad Prism 8 (GraphPad Software, USA). Two-tailed unpaired Student’s *t* test was used to compare two groups of the data, while one-way ANOVA was used to compare multiple groups of data. All data were shown as mean ± SEM. All results shown were representative of at least three independent experiments. A *P* value less than 0.05 is considered as statistically significant.

## Supplementary information

Supplementary Materials

Table S2. Correlation analysis between lipids and metabolic parameters

## Data Availability

All data that support the findings of this study are available from the corresponding author upon reasonable request.

## References

[1] Guzik TJ (2020). COVID-19 and the cardiovascular system: implications for risk assessment, diagnosis, and treatment options. Cardiovasc Res..

[2] Zhou F (2020). Clinical course and risk factors for mortality of adult inpatients with COVID-19 in Wuhan, China: a retrospective cohort study. Lancet.

[3] Chen Y (2020). Clinical characteristics and outcomes of patients with diabetes and COVID-19 in association with glucose-lowering medication. Diabetes Care..

[4] Guo Z, Jiang S, Li Z, Chen S (2021). Metabolic syndrome “interacts” with COVID-19. BIO Integr..

[5] Petrilli CM (2020). Factors associated with hospital admission and critical illness among 5279 people with coronavirus disease 2019 in New York City: prospective cohort study. BMJ.

[6] Wu Z, McGoogan JM (2020). Characteristics of and important lessons from the coronavirus disease 2019 (COVID-19) outbreak in China: summary of a report of 72 314 cases from the Chinese center for disease control and prevention. J. Am. Med. Assoc.

[7] Singh AK, Singh R (2020). Hyperglycemia without diabetes and new-onset diabetes are both associated with poorer outcomes in COVID-19. Diabetes Res. Clin. Pract..

[8] Song S (2021). Association between longitudinal change in abnormal fasting blood glucose levels and outcome of COVID-19 patients without previous diagnosis of diabetes. Front. Endocrinol..

[9] Coppelli A (2020). Hyperglycemia at hospital admission is associated with severity of the prognosis in patients hospitalized for COVID-19: The Pisa COVID-19 Study. Diabetes Care.

[10] Wang S (2020). Fasting blood glucose at admission is an independent predictor for 28-day mortality in patients with COVID-19 without previous diagnosis of diabetes: a multi-centre retrospective study. Diabetologia.

[11] Sardu C (2020). Hyperglycaemia on admission to hospital and COVID-19. Diabetologia.

[12] Wei C (2020). HDL-scavenger receptor B type 1 facilitates SARS-CoV-2 entry. Nat. Metab..

[13] Wang G (2020). Low high-density lipoprotein level is correlated with the severity of COVID-19 patients: an observational study. Lipids Health Dis..

[14] Masana L (2021). Low HDL and high triglycerides predict COVID-19 severity. Sci. Rep..

[15] Coate KC (2020). SARS-CoV-2 cell entry factors ACE2 and TMPRSS2 are expressed in the microvasculature and ducts of human pancreas but are not enriched in β cells. Cell Metab..

[16] Hanley B (2020). Histopathological findings and viral tropism in UK patients with severe fatal COVID-19: a post-mortem study. Lancet Microbe.

[17] Hollstein T (2020). Autoantibody-negative insulin-dependent diabetes mellitus after SARS-CoV-2 infection: a case report. Nat. Metab..

[18] Montefusco L (2021). Acute and long-term disruption of glycometabolic control after SARS-CoV-2 infection. Nat. Metab.

[19] Reiterer M (2021). Hyperglycemia in acute COVID-19 is characterized by adipose tissue dysfunction and insulin resistance. Cell Metab..

[20] Song JW (2020). Omics-driven systems interrogation of metabolic dysregulation in COVID-19 pathogenesis. Cell Metab..

[21] Wu D (2020). Plasma metabolomic and lipidomic alterations associated with COVID-19. Natl Sci. Rev..

[22] Li Y (2021). Multi-platform omics analysis reveals molecular signature for COVID-19 pathogenesis, prognosis and drug target discovery. Signal Transduct. Target Ther..

[23] Overmyer KA (2021). Large-scale multi-omic analysis of COVID-19 severity. Cell Syst..

[24] Shu T (2020). Plasma proteomics identify biomarkers and pathogenesis of COVID-19. Immunity.

[25] Shen B (2020). Proteomic and metabolomic characterization of COVID-19 patient sera. Cell.

[26] Pei R (2020). Host metabolism dysregulation and cell tropism identification in human airway and alveolar organoids upon SARS-CoV-2 infection. Protein Cell.

[27] Yang J (2020). Prevalence of comorbidities and its effects in patients infected with SARS-CoV-2: a systematic review and meta-analysis. Int. J. Infect. Dis..

[28] Lubec G, Afjehi-Sadat L (2007). Limitations and pitfalls in protein identification by mass spectrometry. Chem. Rev..

[29] Blanco-Melo D (2020). Imbalanced host response to SARS-CoV-2 drives development of COVID-19. Cell.

[30] Troncone E (2021). Low frequency of acute pancreatitis in hospitalized COVID-19 patients. Pancreas.

[31] Pezzilli R (2021). Patients with coronavirus disease 2019 interstitial pneumonia exhibit pancreatic hyperenzymemia and not acute pancreatitis. Pancreas.

[32] Papayannopoulos V (2018). Neutrophil extracellular traps in immunity and disease. Nat. Rev. Immunol..

[33] Wang Q (2014). Myeloperoxidase deletion prevents high-fat diet-induced obesity and insulin resistance. Diabetes.

[34] Chai W (2019). Inhibiting myeloperoxidase prevents onset and reverses established high-fat diet-induced microvascular insulin resistance. Am. J. Physiol. Endocrinol. Metab..

[35] Mughal A, O’Rourke ST (2018). Vascular effects of apelin: mechanisms and therapeutic potential. Pharm. Ther..

[36] Gourdy P (2018). Apelin administration improves insulin sensitivity in overweight men during hyperinsulinaemic-euglycaemic clamp. Diabetes Obes. Metab..

[37] Zhang C (2011). Myostatin-deficient mice exhibit reduced insulin resistance through activating the AMP-activated protein kinase signalling pathway. Diabetologia.

[38] Feldman BJ, Streeper RS, Farese RV, Yamamoto KR (2006). Myostatin modulates adipogenesis to generate adipocytes with favorable metabolic effects. Proc. Natl Acad. Sci. USA.

[39] Chong JA (1995). REST: a mammalian silencer protein that restricts sodium channel gene expression to neurons. Cell.

[40] Martin D (2008). Functional significance of repressor element 1 silencing transcription factor (REST) target genes in pancreatic beta cells. Diabetologia.

[41] Tirosh A (2019). The short-chain fatty acid propionate increases glucagon and FABP4 production, impairing insulin action in mice and humans. Sci Transl Med.

[42] Heimann E (2016). Branched short-chain fatty acids modulate glucose and lipid metabolism in primary adipocytes. Adipocyte.

[43] Chinese Diabetes Society. (2018). Guidelines for the prevention and control of type 2 diabetes in China (2017 Edition). Chin. J. Practical Intern. Med..

[44] Lisheng Liu (2019). 2018 Chinese guidelines for the management of hypertension. Chin. J. Cardiovasc. Med..

[45] Junren Chu (2017). 2016 Chinese guideline for the management of dyslipidemia in adults. Chin. J. Health Manag..

[46] Jiangao Fan, Lai Wei, Hui Zhuang (2018). Guidelines of prevention and treatment for nonalcoholic fatty liver disease: a 2018 update. Infect. Dis. Inf..

[47] Li Z (2020). Resveratrol promotes white adipocytes browning and improves metabolic disorders in Sirt1-dependent manner in mice. FASEB J..

[48] Onay-Besikci A, Suzmecelik E, Ozcelikay AT (2012). Carvedilol suppresses fatty acid oxidation and stimulates glycolysis in C2C12 cells. Can. J. Physiol. Pharmacol..

[49] Zeng J, Li G (2018). TFmapper: a tool for searching putative factors regulating gene expression using ChIP-seq data. Int. J. Biol. Sci..

[50] Ochsner SA (2019). The Signaling Pathways Project, an integrated ‘omics knowledgebase for mammalian cellular signaling pathways. Sci. Data..

