# Supplementary material for: COVID-19 induces new-onset insulin resistance and lipid metabolic dysregulation via regulation of secreted metabolic factors
Source: Signal Transduct Target Ther. 2021 Dec 16;6:427. doi: 10.1038/s41392-021-00822-x (PMC8674414; doi:10.1038/s41392-021-00822-x)
Supplement: Supplementary file 1 — Supplementary Materials [file 41392_2021_822_MOESM1_ESM.docx]

Supplementary Materials for

COVID-19 induces new-onset insulin resistance and lipid metabolic dysregulation via regulation of secreted metabolic factors

Xi He^1#^, Chenshu Liu^2,3#^, Jiangyun Peng^4,5#^, Zilun Li^2,3#^, Fang Li^1,6#^, Jian Wang^1^, Ao Hu^7,8^, Meixiu Peng^3^, Kan Huang^2,3^, Dongxiao Fan^2,3^, Na Li^2,3^, Fuchun Zhang^1^, Weiping Cai^1^, Xinghua Tan^1^, Zhongwei Hu^1^, Xilong Deng^1^, Yueping Li^1^, Xiaoneng Mo^1^, Linghua Li^1^, Yaling Shi^1^, Li Yang^1^, Yuanyuan Zhu^1^, Yanrong Wu^1^, Huichao Liang^1^, Baolin Liao^1^, Wenxin Hong^1^, Ruiying He^1^, Jiaojiao Li^1^, Pengle Guo^1^, Youguang Zhuo^1^, Lingzhai Zhao^1^, Fengyu Hu^1^, Wenxue Li^9^, Wei Zhu^9^, Zefeng Zhang^4,5^, Zeling Guo^3^, Wei Zhang^10^, Xiqiang Hong^10^, Weikang Cai^11^, Lei Gu^12^, Ziming Du^13^, Yang Zhang^14^, Jin Xu^15^, Tao Zuo^16^, Kai Deng^7,8^, Li Yan^17^, Xinwen Chen^18,19^*, Sifan Chen^4,5^*, Chunliang Lei^1^*

*Corresponding authors: Xinwen Chen, Guangzhou Regenerative Medicine and Health-Guangdong Laboratory (GRMH-GDL), Key Laboratory of Regenerative Biology of the Chinese Academy of Sciences and Guangdong Provincial Key Laboratory of Stem Cell and Regenerative Medicine, Guangzhou Institutes of Biomedicine and Health, Chinese Academy of Sciences, Guangzhou 510530, China. E-mail: chen_xinwen@gibh.ac.cn

Sifan Chen, Guangdong Provincial Key Laboratory of Malignant Tumor Epigenetics and Gene Regulation, Guangdong-Hong Kong Joint Laboratory for RNA Medicine; Medical Research Center, Sun Yat-Sen Memorial Hospital, Sun Yat-Sen University, Guangzhou 510320, China. E-mail: chensf26@mail.sysu.edu.cn

Chunliang Lei, Guangzhou Eighth People’s Hospital, Guangzhou Medical University, Guangzhou 510060, China. E-mail: gz8hlcl@126.com

**This PDF file includes:**

Figures. S1 to S6

Table S1


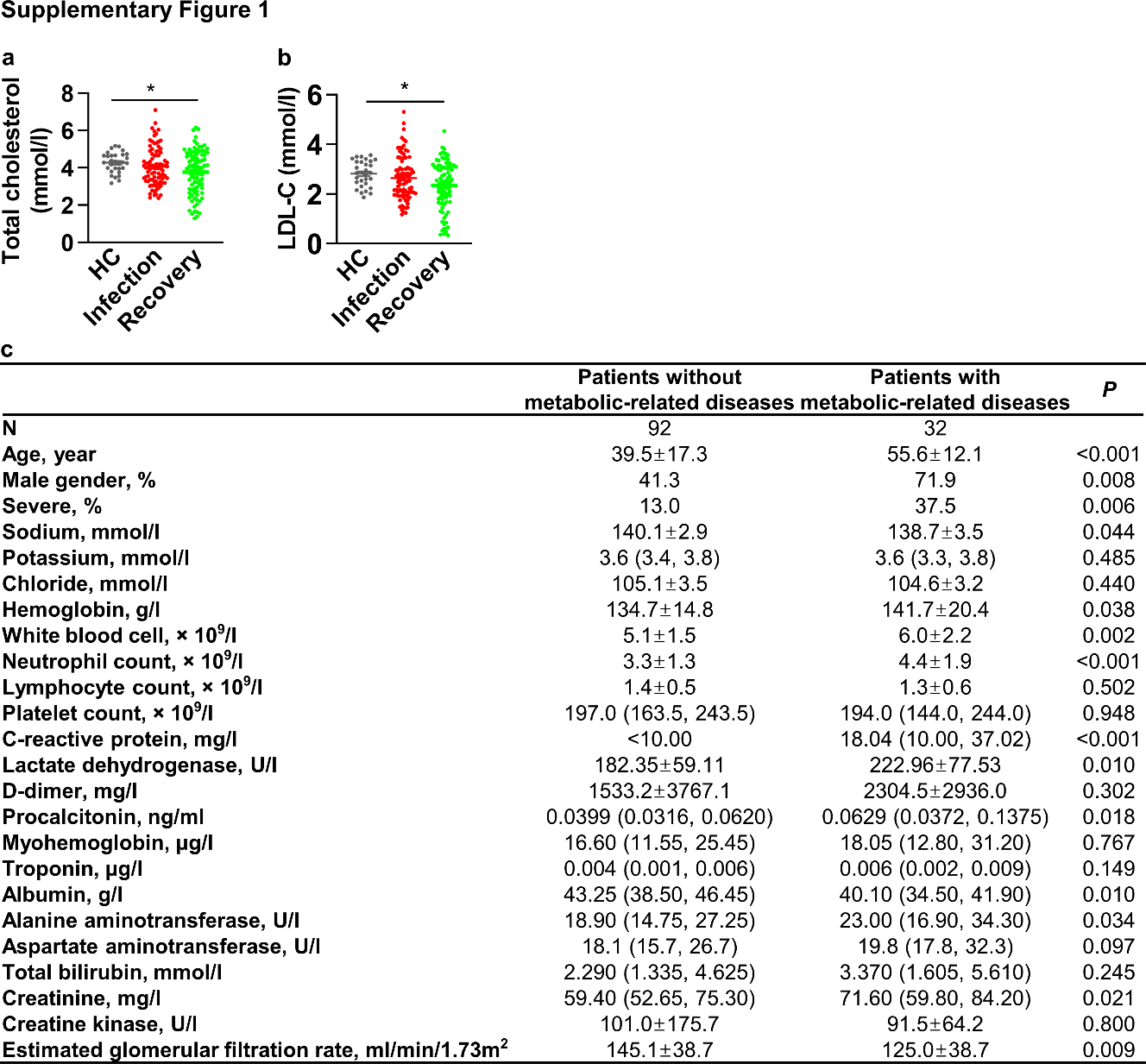


Figure. S1. Glucose and lipid metabolic dysregulation in sera of COVID-19 patients, Related to Fig. 1.

a, Blood total cholesterol in healthy control group and COVID-19 infection and recovery group (HC, healthy control, n = 30; COVID-19 infection and recovery, n = 92). b, Blood low density lipoprotein cholesterol (LDL-C) in healthy control group and COVID-19 infection and recovery group (HC, n = 30; COVID-19 infection and recovery, n = 92). c, Basic characteristics, biochemical and metabolic parameters in patients without metabolic-related diseases and patients with metabolic-related diseases were shown. Error bars represent SEM. *p < 0.05.


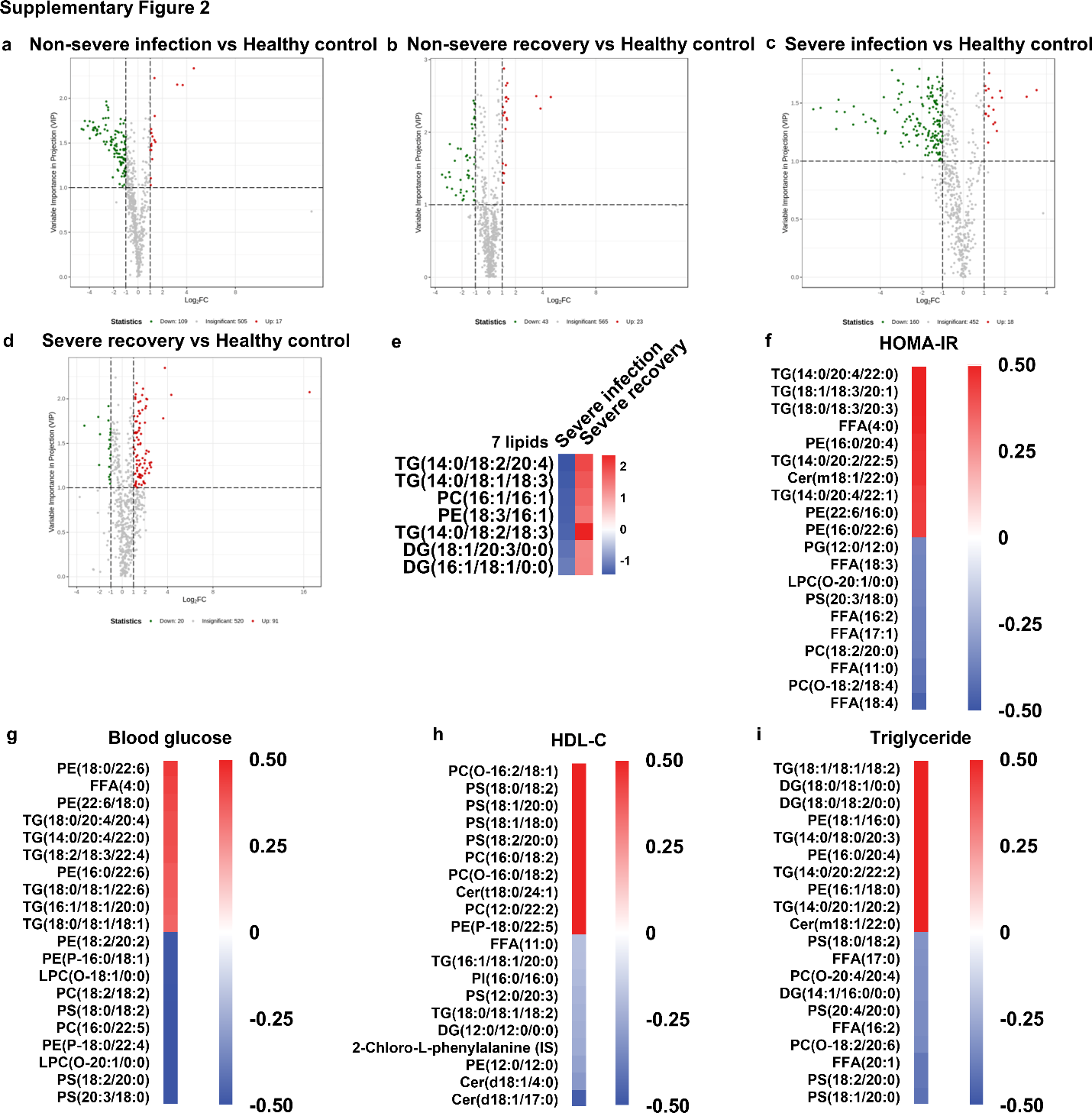


Figure. S2. Lipidomics profiling in sera of COVID-19 patients, Related to Fig. 2.

a, Volcano plot of differential lipids in comparison of non-severe infection group against healthy control group. b, Volcano plot of differential lipids in comparison of non-severe recovery group against healthy control group. c, Volcano plot of differential lipids in comparison of severe infection group against healthy control group. d, Volcano plot of differential lipids in comparison of severe recovery group against healthy control group. e, The heatmap was shown to represent the alterations of differential lipids in severe infection phase and recovery phase against healthy control, respectively. f-i, The heatmap was shown to represent top 10 positive- and negative-correlated lipids between all 631 identified lipids and HOMA-IR, blood glucose, HDL-C and triglyceride, respectively.


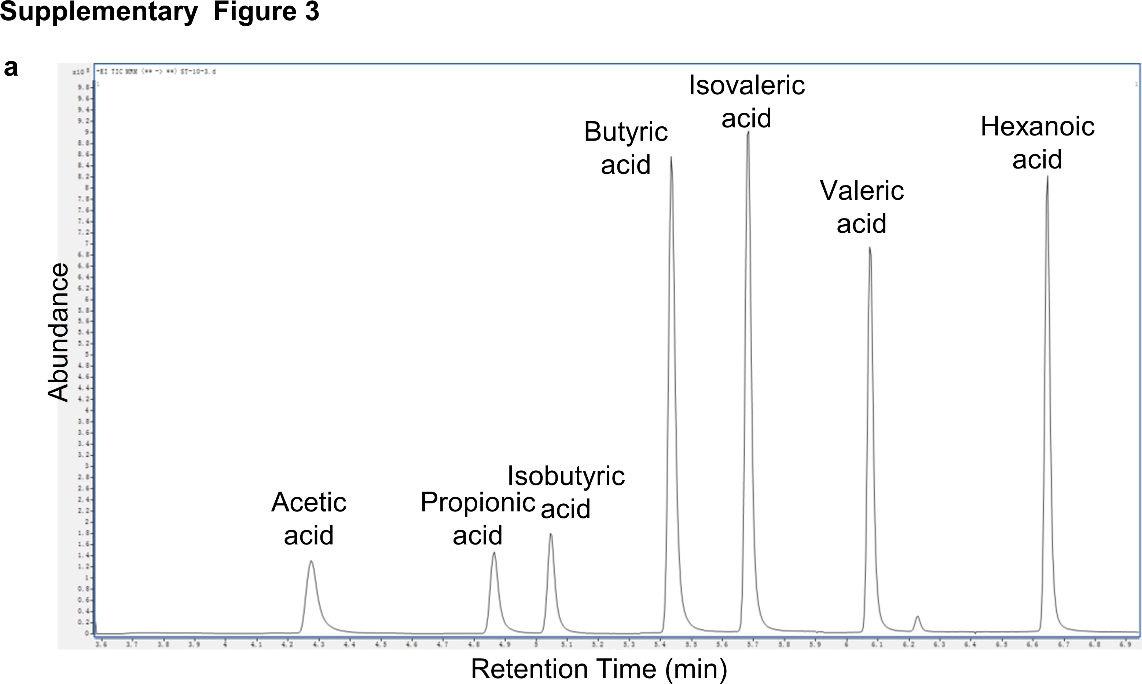


Figure. S3. The short chain fatty acids profiling in sera of COVID-19 patients, Related to Fig. 3.

a, Total ionic chromatography of acetic acid, propionic acid, isobutyric acid, butyric acid, isovaleric acid, valeric acid and hexanoic acid.


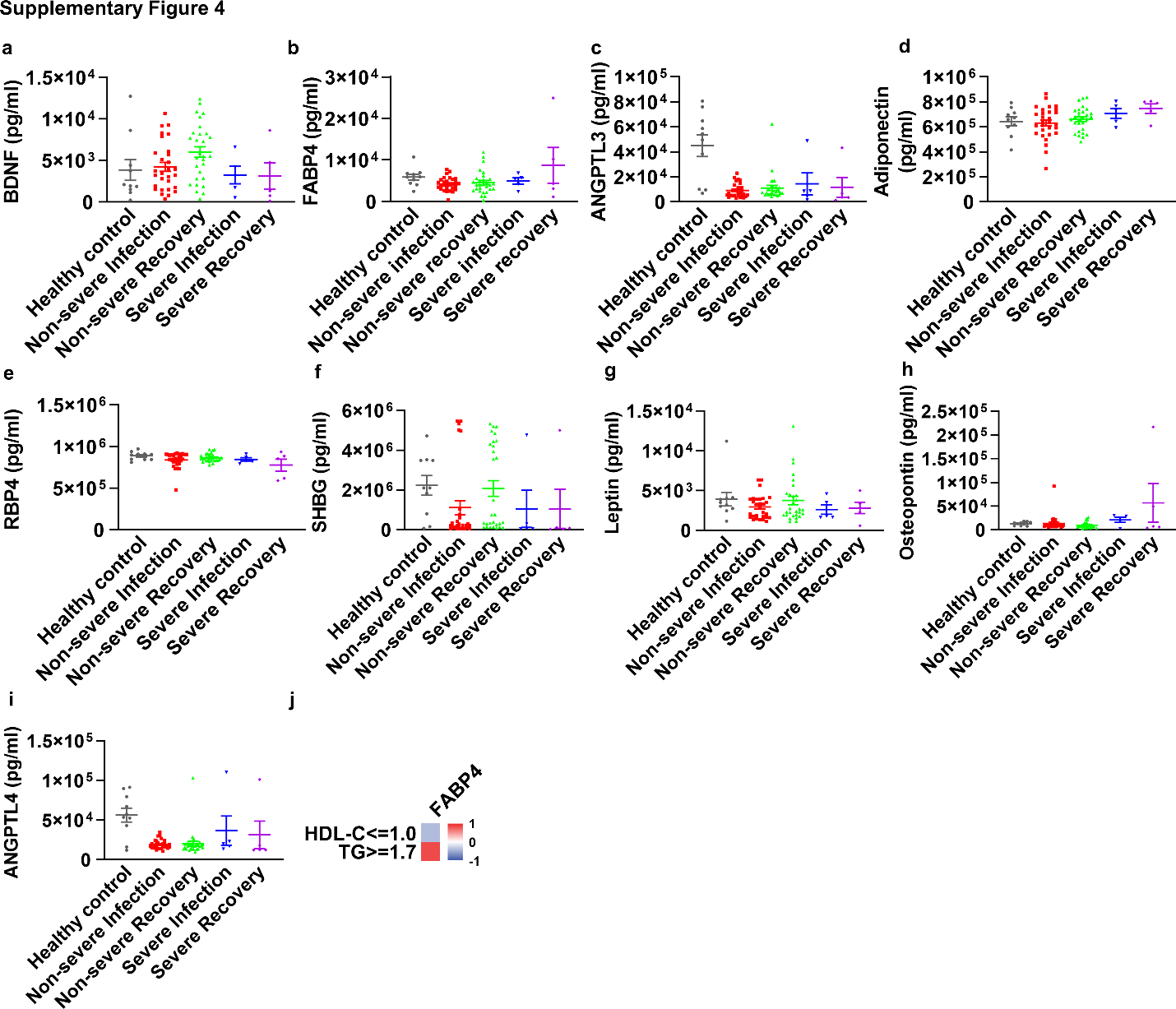


Figure. S4. The measurements of secreted metabolic factors in serum sera of COVID-19 patients, Related to Fig. 4.

a-i, Concentration of sera metabolic factors in healthy control (n = 10), non-severe infection (n = 29), non-severe recovery group (n = 29), severe infection (n = 5), and severe recovery group (n = 5) were shown. j, The heatmap was shown to represent the correlation between FABP4 and metabolic parameters. Data were shown as R-square


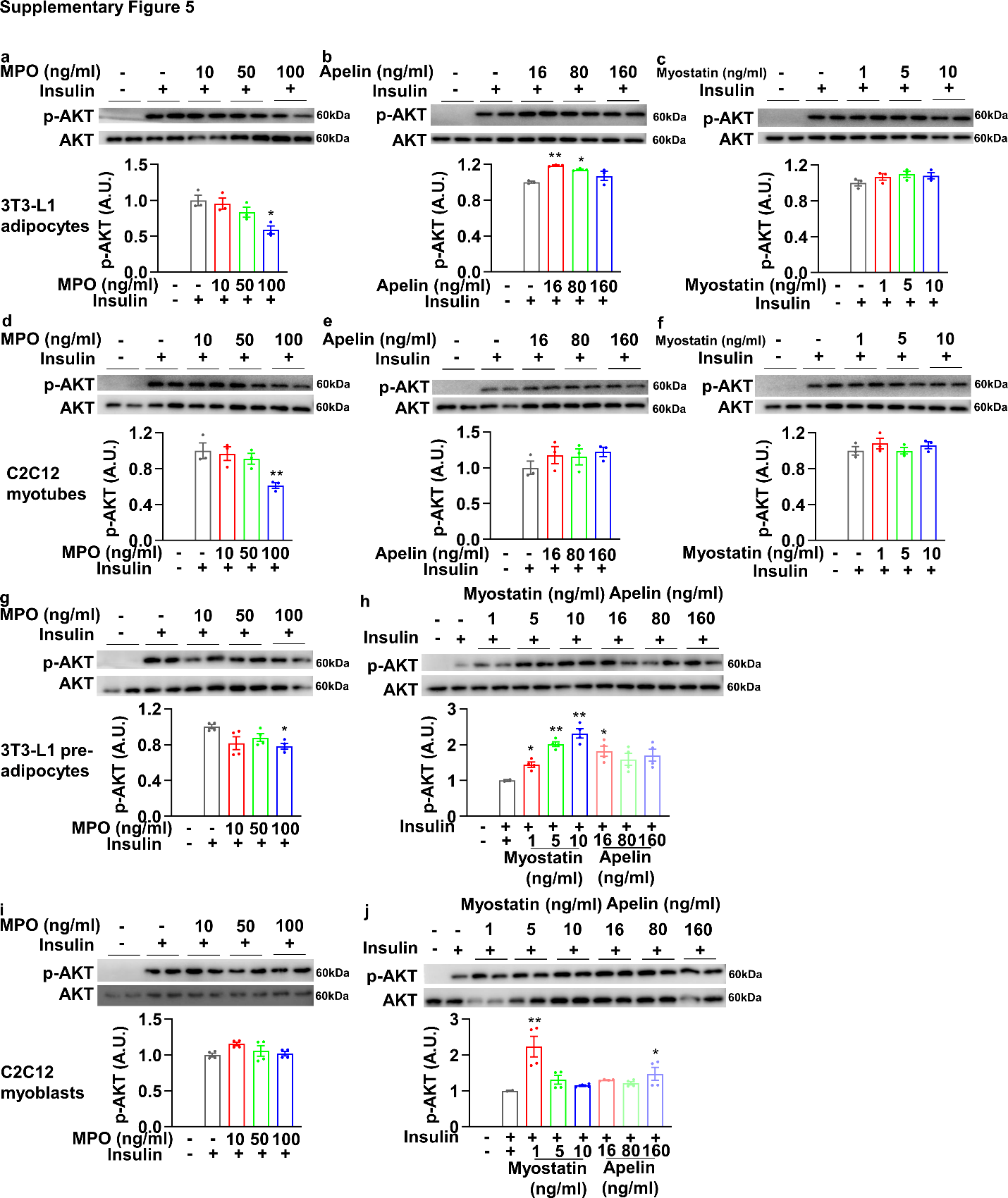


Figure. S5. The metabolic regulation of MPO, apelin, and myostatin in vitro, Related to Fig. 5.

**a-c,** 3T3-L1 adipocytes, **d-f**, C2C12 myotubes, **g-h**, 3T3-L1 pre-adipocytes and **i-j** C2C12 myoblasts were treated with myeloperoxidase (MPO), apelin or myostatin for 24 h following starvation with DMEM medium for 1 h, then 10 nM insulin was administrated for 15 min and subjected to western blot (n = 3, quantifications were shown below the blots).


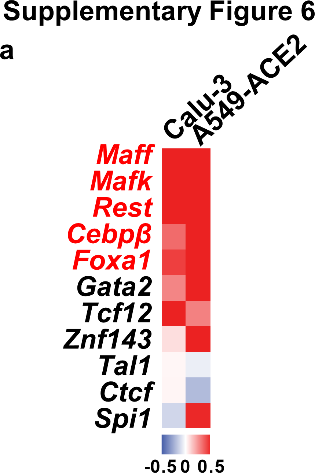


Figure. S6. MPO, apelin and myostatin were regulated by transcription factor REST, Related to Fig. 6.

a, The heatmap was shown to represent the alterations of 11 transcription factors upon SARS-CoV-2 infection in Calu-3 cells or A549 cells with overexpression of ACE2 (A549-ACE2). Data were shown as log2 fold change.

Table S1. Oligos used in this study, related to Methods section.

| Oligo name | Sequence (5’–3’) | | Purpose |
| --- | --- | --- | --- |
| Mouse *G6pc* | Forward | TGGCTTTTTCTTTCCTCGAA | qPCR |
| Mouse *G6pc* | Reverse | TCGGAGACTGGTTCAACCTC | qPCR |
| Mouse *Pck1* | Forward | GGCGATGACATTGCCTGGATGA | qPCR |
| Mouse *Pck1* | Reverse | TGTCTTCACTGAGGTGCCAGGA | qPCR |
| Mouse *Pfkl* | Forward | ATCCCAACTCGAGATTCTGC | qPCR |
| Mouse *Pfkl* | Reverse | CATGGCTGCTCCTACAAACA | qPCR |
| Mouse *Srebp1* | Forward | CGACTACATCCGCTTCTTGCAG | qPCR |
| Mouse *Srebp1* | Reverse | CCTCCATAGACACATCTGTGCC | qPCR |
| Mouse *Fasn* | Forward | CACAGTGCTCAAAGGACATGCC | qPCR |
| Mouse *Fasn* | Reverse | CACCAGGTGTAGTGCCTTCCTC | qPCR |
| Mouse *Scd* | Forward | GCAAGCTCTACACCTGCCTCTT | qPCR |
| Mouse *Scd* | Reverse | CGTGCCTTGTAAGTTCTGTGGC | qPCR |
| Human *Tnfa* | Forward | GAGGCGCTCCCCAAGAAGAC | qPCR |
| Human *Tnfa* | Reverse | CAGGCTTGTCACTCGGGGTT | qPCR |
| Human *Il-6* | Forward | GCAAGGGTCTGGTTTCAGCCT | qPCR |
| Human *Il-6* | Reverse | TCGCTCCCTCTCCCTGTAAGT | qPCR |
| Human *Vcam1* | Forward | GATTCTGTGCCCACAGTAAGGC | qPCR |
| Human *Vcam1* | Reverse | TGGTCACAGAGCCACCTTCTTG | qPCR |
| Human *Cd36* | Forward | GGCTGTGACCGGAACTGTG | qPCR |
| Human *Cd36* | Reverse | AGGTCTCCAACTGGCATTAG | qPCR |
| Human *Glut1* | Forward | TTGCAGGCTTCTCCAACTGGAC | qPCR |
| Human *Glut1* | Reverse | CAGAACCAGGAGCACAGTGAAG | qPCR |
| Human *Foxa1* | Forward | ACCAGCGACTGGAACAGCTAC | qPCR |
| Human *Foxa1* | Reverse | GCCGCTCGTAGTCATGGTGT | qPCR |
| Human *Maff* | Forward | CCACAACAAAACTCAGCGCA | qPCR |
| Human *Maff* | Reverse | GTGTTCTCGCTCAGCTCTCG | qPCR |
| Human *Rest* | Forward | CCGTGGGGGAGTGAAGTTCG | qPCR |
| Human *Rest* | Reverse | TGTTTGGCCGGAACCAGTCC | qPCR |
| Human *Mafk* | Forward | GCCCCACAACAAAACTCAGCG | qPCR |
| Human *Mafk* | Reverse | TGTTCTCGCTCAGCTCTCGC | qPCR |
| Human *Cebpβ* | Forward | TACACGGGACTGACGCAACC | qPCR |
| Human *Cebpβ* | Reverse | TGCCCCCAAAAGGCTTTGTA | qPCR |
| Human *Mpo* | Forward | CCCCTGCCAAGCTGAATCGT | qPCR |
| Human *Mpo* | Reverse | GGCGTGCCATACTGCTCCAT | qPCR |
| Human *Apln* | Forward | GCCCCCATAACTGGCAGCAT | qPCR |
| Human *Apln* | Reverse | AGGTCCGGTCAACACGAAGG | qPCR |
| Human *Mstn* | Forward | TCGGCGAGGAAATGAAAGCG | qPCR |
| Human *Mstn* | Reverse | GCACCGTTGGCATGGATTGT | qPCR |
| pcDNA 3×FLAG-FOXA1 | Forward | CTTGGTACCGAGCTCGGATCCG  CCACCATGTTAGGAACTGTGAAGATGG | Cloning |
|  | Reverse | GAAGGGCCCTCTAGACTCGAGGGAAGTGTTTAGGACGGGT |  |
| pcDNA 3×FLAG-MAFF | Forward | CTTGGTACCGAGCTCGGATCCGCC  ACCATGTCTGTGGATCCCCTATCCA | Cloning |
|  | Reverse | GAAGGGCCCTCTAGACTCGAGGGAGCAGGAGGCCGGGC |  |
| pcDNA 3×FLAG-REST | Forward | CTTGGTACCGAGCTCGGATCCGCCA  CCATGGCCACCCAGGTAATGGG | Cloning |
|  | Reverse | GAAGGGCCCTCTAGACTCGAGCTCCTGCCCTTGAGCTGC |  |
| siFOXA1 |  | TCCCGGTCAGCAACATGAA | siRNA |
| siMAFF |  | CCGCACACTCAAAAACCGT | siRNA |
| siREST |  | CTGTGATCGTTCTTTCAGA | siRNA |
